# Oxalate deposition in renal allograft biopsies within 3 months after transplantation is associated with allograft dysfunction

**DOI:** 10.1371/journal.pone.0214940

**Published:** 2019-04-16

**Authors:** Malou L. H. Snijders, Dennis A. Hesselink, Marian C. Clahsen-van Groningen, Joke I. Roodnat

**Affiliations:** 1 Department of Pathology, Erasmus MC, University Medical Center Rotterdam, Rotterdam, The Netherlands; 2 Rotterdam Transplant Group, Erasmus MC, University Medical Center Rotterdam, Rotterdam, The Netherlands; 3 Department of Internal Medicine, Division of Nephrology and Transplantation, University Medical Center Rotterdam, Rotterdam, The Netherlands; University of Toledo, UNITED STATES

## Abstract

**Background:**

Calcium oxalate (CaOx) deposition in the kidney may lead to loss of native renal function but little is known about the prevalence and role of CaOx deposition in transplanted kidneys.

**Methods:**

In patients transplanted in 2014 and 2015, all for-cause renal allograft biopsies obtained within 3 months post-transplantation were retrospectively investigated for CaOx deposition. Additionally, all preimplantation renal biopsies obtained in 2000 and 2001 were studied.

**Results:**

In 2014 and 2015, 388 patients were transplanted, of whom 149 had at least one for-cause renal biopsy. Twenty-six (17%) patients had CaOx deposition. In the population with CaOx deposition: Patients had significantly more often been treated with dialysis before transplantation (89 vs. 64%; p = 0.011); delayed graft function occurred more frequently (42 vs. 23%; p = 0.038); and the eGFR at the time of first biopsy was significantly worse (21 vs. 29 ml/min/1.73m^2^; p = 0.037). In a multivariate logistic regression analysis, eGFR at the time of first biopsy (OR 0.958, 95%-Cl: 0.924–0.993, p = 0.019), dialysis before transplantation (OR 4.868, 95%-Cl: 1.128–21.003, p = 0.034) and the time of first biopsy after transplantation (OR 1.037, 95%-Cl: 1.013–1.062, p = 0.002) were independently associated with CaOx deposition. Graft survival censored for death was significantly worse in patients with CaOx deposition (p = 0.018). In only 1 of 106 preimplantation biopsies CaOx deposition was found (0.94%).

**Conclusion:**

CaOx deposition appears to be primarily recipient-derived and is frequently observed in for-cause renal allograft biopsies obtained within 3 months post-transplantation. It is associated with inferior renal function at the time of biopsy and worse graft survival.

## Introduction

Oxalic acid is a small decarboxylate ion (C_2_O_4_) and is the end-product of many metabolic pathways. Oxalic acid is eliminated through free glomerular filtration and secretion by the proximal tubule [1]. The plasma concentration of oxalic acid is determined by the balance between dietary intake, intestinal absorption, endogenous production and renal excretion [2]. Normal plasma concentrations are below 5 μmol/l [3–4].

Hyperoxaluria, defined as an excessive urinary excretion of oxalic acid, can be classified as primary or secondary hyperoxaluria. Primary hyperoxaluria is caused by rare autosomal recessive disorders which cause an excessive production of oxalic acid [5–7]. In primary hyperoxaluria, persistently elevated plasma oxalic acid concentrations cause CaOx deposition in the kidney which leads to permanent loss of renal function. Combined liver-kidney transplantation is recommended in these cases [5–6, 8].

Secondary hyperoxaluria is a more common disorder which can be caused by enteric conditions and increased dietary intake of oxalate. In enteric hyperoxaluria, increased absorption of oxalic acid occurs. These are mostly patients with malabsorption as a result of e.g. small bowel resections, pancreatic insufficiency or gastric bypass. A high intake of oxalic acid-containing food puts these patients at risk of renal stones or CaOx deposition in their kidneys [9–11]. In addition, studies suggest a contribution of dietary oxalate in renal CaOx deposition in patients without enteric conditions as well [12–15].

When the GFR drops below 30–40 ml/min/1.73 m^2^ oxalic acid elimination by the kidneys is impaired and the plasma concentration rises [5]. In patients with end-stage renal disease (ESRD), high plasma oxalic acid concentrations, that may be up to 10 times above normal levels, may occur [16–18].

Neither hemo- nor peritoneal dialysis can remove sufficient amounts of oxalic acid to normalize the oxalic acid plasma concentrations in patients with ESRD, although it is suggested that the clearance of oxalic acid by hemodialysis exceeds that of peritoneal dialysis [19–20]. The plasma oxalic acid concentration can be reduced by at least 60% following a single hemodialysis session. However, it returns to pre-dialysis levels within 48 hours [21–22].

Plasma oxalic acid concentrations generally normalize within days to weeks after a successful kidney transplantation [23–25]. However, in the first few days after transplantation, a large amount of oxalate is excreted [25]. The urine becomes supersaturated with oxalate when concentrations >30 μmol/L are reached, and this may result in the formation of CaOx crystals, which can deposit in the renal tubules [5]. Therefore, it can be expected that CaOx deposition is mainly formed in the early post transplantation period, while it probably is less prevalent in the late post transplantation period. However, it is unknown whether this phenomenon affects renal transplant outcome.

The purpose of the present study was to examine if 1) CaOx deposition is associated with kidney allograft dysfunction in the immediate post-operative phase; 2) CaOx deposition is associated with inferior graft survival; and 3) CaOx deposition in the transplanted kidney is recipient- rather than donor-derived. To investigate this, the incidence of CaOx deposition was studied in for-cause kidney allograft biopsies obtained within the first 3 months after transplantation, as well as in a series of preimplantation renal biopsies.

### Patients and methods

All patients transplanted between January 2014 and December 2015 in the Erasmus MC, University Medical Center Rotterdam, Rotterdam, The Netherlands, were included. The databases of the Departments of Renal Transplantation and Pathology were searched to identify all for-cause renal allograft biopsies performed in these patients within the first 3 months. No biopsies were performed for the sake of the present study. Clinical and demographic data of these patients were collected. These included: age at transplantation, type of dialysis (hemodialysis or peritoneal dialysis) and time on dialysis, donor age and type (living or deceased), cold ischemia time (CIT), first warm ischemia time (1^st^ WIT), second warm ischemia time (2^nd^ WIT) and delayed graft function (DGF). For those patients with a for-cause renal biopsy we collected: time to first biopsy, histologic diagnosis (acute rejection/acute tubular necrosis (ATN)/other), best estimated GFR (eGFR) before biopsy and eGFR at time of the for-cause biopsy. DGF was defined as the need for dialysis during the first week after transplantation [26–27].

In addition, all preimplantation renal transplant biopsies (time zero, t0) obtained in 2000 and 2001 were retrospectively searched (in the database of the Department of Pathology) and re-analyzed for the presence of CaOx, as described below. Both living and deceased donor t0 biopsies were included.

### Histological evaluation

All for-cause and t0 biopsies were processed according to standardized routine diagnostic practice. Light microscopy was performed on 3 μm sections of formalin-fixed paraffin embedded tissue that was stained by hematoxylin and eosin (H&E). CaOx deposition was analyzed on these sections using polarized light by two pathologists (MS and MCvG) who were blinded to patient information. In case of disagreement, consensus regarding the presence of CaOx deposition was reached. CaOx deposition was scored by counting the total number of oxalate depositions in each biopsy. Biopsies were defined as positive for CaOx when ≥1 CaOx deposits were found within the tubular lumen, tubular epithelial cells and/or interstitial space. The surface area of the biopsies with CaOx deposition was measured using the software program ImageJ and expressed as mm^2^ [28–29]. The amount of CaOx depositions per mm^2^ was calculated as described in the study by Bagnasco *et al* [30].

### Statistical analysis

SPSS Statistics version 23.0 was used for all statistical analyses (IBM SPSS Statistics for Windows. Armonk, NY: IBM Corp.). For comparison of continuous variables, the one-way ANOVA-test was used. Categorical variables were compared using the Chi-square test. Data were presented as mean ± standard deviation (SD) for continuous variables and as percentages for categorical variables. Differences with a (two sided) p-value of less than or equal to 0.05 were considered statistically significant. Univariate and multivariate logistic regression analysis was used to evaluate which variables are independently associated with CaOx deposition.

Graft survival censored for death was compared using Kaplan Meier analysis and log rank test.

### Medical ethics

The study protocol was consistent with professional guidelines; non WMO compliant research is research not residing under the Dutch law on medical research with patients and is regulated by the Dutch Code of Conduct (Federa). The local medical ethics committee of the Erasmus MC, University Medical Center Rotterdam, Rotterdam, The Netherlands, approved that our study is exempt from the requirement for approval (MEC-2018-1580). Informed consent to the use of medical record data and rest material was waived in accordance with the Dutch regulations. All data were anonymized prior to analysis.

## Results

### Patient characteristics

A total of 388 patients were transplanted in the period studied (2014 and 2015). Of these 388 patients, 77 (19.8%) had DGF. At least one for-cause renal transplant biopsy was performed in 149 patients (38%) and these patients were included in the present study. The characteristics of these patients are depicted in Table 1. In the period studied, 1 patient with primary hyperoxaluria underwent a combined kidney and liver transplantation. Interestingly, a renal allograft biopsy of this patient 9 months after transplantation did not show CaOx deposition. Renal function of this patient remains good 3 years after transplantation. Three patients with enteric hyperoxaluria as the primary kidney disease were transplanted in the period studied. Of these, one was not biopsied during the first year after transplantation. The other two were biopsied in the first 3 months after transplantation. In one patient, the renal biopsy showed CaOx deposition, whereas in the other patient no CaOx deposition was seen. Transplant function remains good in all patients 3.5 years after transplantation. All 4 patients described above had received oxalate lowering treatment pre- and post-transplantation [31].

**Table 1:** Characteristics of patients with and without CaOx depositions in their for-cause biopsy.

Parameters	All	No oxalate depositions	Oxalate depositions	P-value
**N (%)**	149	123 (83)	26 (17)	
**Recipient age (years)**	54±14	55±14	52±15	0.409
**Time on dialysis (months)**	19±25	18±26	23±21	0.833
**RRT before transplantation**				0.011
** HD/PD**	102 (68.5)	79 (64)	23(89)	
** None**	47 (31.5)	44 (36)	3 (11)	
**Donor age (years)**	55±14	55±14	55±13	0.934
**Living donor (%)**	97 (65)	84 (68)	13 (50)	0.062
**CIT (min)**	362±321	346±316	436±344	0.201
**1st WIT (min)**	4.8±5.7	4.8±5.4	5.1±7.2	0.777
**2nd WIT (min)**	21.1±6	20.9±6	21.8±6	0.493
**Time of first biopsy** **(days after Tx)**	20±24	19±23	26±29	0.123
**Diagnosis (%)**				0.411
** ATN**	36 (24)	29 (23)	7 (27)	
** Rejection**	69 (46)	55 (45)	14 (54)	
** Other/none**	44 (30)	39 (32)	5 (19)	
**DGF**	39 (26)	28 (23)	11 (42)	0.038
**Best eGFR before biopsy (ml/min/1.73m**^**2**^**)**	36±24	37±23	33±26	0.398
**eGFR at first biopsy (ml/min/1.73m**^**2**^**)**	27±17	29±17	21±17	0.037

RRT, renal replacement therapy; HD, hemodialysis; PD, peritoneal dialysis; CIT, cold ischemia time; WIT, warm ischemia time; ATN, acute tubular necrosis; DGF, delayed graft function; eGFR, estimated glomerular filtration rate.

A total of 196 biopsies from 149 patients was analyzed, with a median of 1 biopsy per patient (range 1–3). Patients’ age ranged from 19 to 79 years (mean 54 ± 14 years). Ninety-seven patients (65%) received a graft from a living donor and 52 patients (35%) from a deceased donor. The histological diagnosis was rejection in 69 patients (46%), ATN in 36 patients (24%), and ‘‘other” in the remaining 44 (30%) patients (Table 1).

### CaOx deposition in the renal allograft biopsy

Twenty-six patients (17%) showed CaOx deposition in their for-cause biopsy obtained within 3 months after transplantation. In 111 patients only 1 biopsy was performed and CaOx deposition was observed in 14 of them. Thirty-eight patients had more than 1 biopsy within 3 months after renal transplantation and CaOx deposition was observed in 12 of them; in 3 patients CaOx deposition was found in all the biopsies, in 3 patients CaOx deposition was observed in the first biopsy but not in the second (or third) biopsy. Six patients showed CaOx deposition in their repeat biopsy only.

**Fig 1:**
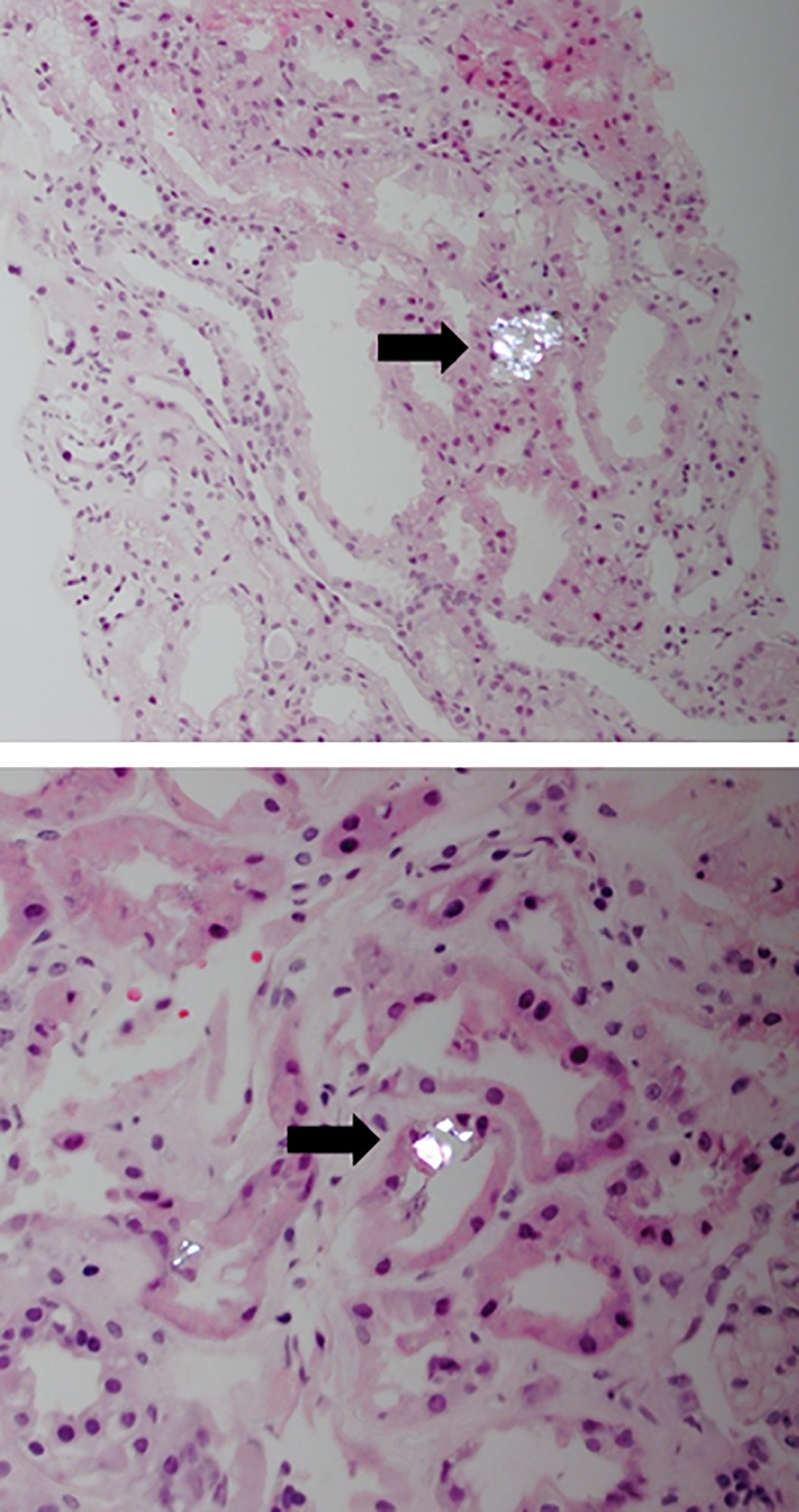
Examples of an H&E staining of a renal allograft biopsy examined by polarized light showing CaOx deposition (arrow) (magnification 10x and 20x respectively).

The CaOx crystals were translucent in H&E sections and birefringent structures were seen using polarized light (Fig 1). CaOx deposits were mostly observed in the lumen and within the epithelial cells of the tubules in the cortex. Occasionally, CaOx deposits were observed in the medulla. The mean surface of the biopsies containing CaOx deposition was 7,9 ± 2,3 mm^2^ (range 4,1–12,6 mm^2^) and the total amount of CaOx deposition varied between 1 and 33 deposits per biopsy (mean 6 ± 7 CaOx deposits per biopsy). The density of CaOx deposition varied between 0,1 and 4,4 deposits/ mm^2^ (mean density 0,8 ± 1,0 deposits/ mm^2^).

Table 1 shows the patient characteristics of patients with and without CaOx depositions. Significantly more patients with CaOx deposition had renal replacement therapy (RRT) (hemodialysis or peritoneal dialysis) before transplantation (p = 0.011). CaOx depositions were more frequently observed in the population with RRT compared to those who were transplanted pre-emptively but the difference was not significant for peritoneal dialysis and hemodialysis separately (p = 0.051). Time on dialysis was not significantly different between those patients with and those without CaOx depositions (p = 0.88).

The best eGFR before biopsy was not significantly different between patients with and those without CaOx deposition. However, eGFR at time of the first biopsy was significantly lower in patients with CaOx deposition (p = 0.037). DGF occurred more frequently in patients with CaOx deposition (p = 0.038). No histological diagnosis (rejection, ATN or other) prevailed in the CaOx group. All other variables were not significantly different between the groups. In addition, no significant association was observed between CaOx density (≥ 0,8 deposits/ mm^2^ *vs*. < 0,8 deposits/ mm^2^) and eGFR at time of first biopsy (p = 0.297) and between CaOx density and DGF (p = 0.190).

As there were 26 patients with CaOx deposition in their allograft biopsy, the number of variables that could be analyzed at the same time in the regression analysis was limited. Three variables showed a significant influence on CaOx deposition in the univariate analysis: eGFR at the time of first biopsy (odds ratio (OR) 0.971, 95%-Cl: 0.945–0.999, p = 0.040), DGF (OR 2.488, 95%-Cl: 1.027–6.028, p = 0.043) and RRT before transplantation (OR 4.270, 95%-Cl: 1.213–15.029, p = 0.024). A stepwise multivariate analysis with backward elimination was performed, adding all variables with p < 0.2 in the univariate analysis. Eventually, three variables were independently associated with CaOx deposition in the multivariate analysis; eGFR at the time of first biopsy (OR 0.958, 95%-Cl: 0.924–0.993, p = 0.019), RRT before transplantation (OR 4.868, 95%-Cl: 1.128–21.003, p = 0.034) and time of first biopsy after transplantation (OR 1.037, 95%-Cl: 1.013–1.062, p = 0.002).

### CaOx in renal transplantectomies within 3 months after transplantation

Of all patients transplanted between January 2014 and December 2015, a total of 8 transplantectomies were performed within 3 months after transplantation. In 3 of them CaOx deposition was found. Of these 3 patients 1 had a prior allograft biopsy that showed CaOx deposition, 1 had an allograft biopsy without CaOx deposition and 1 did not have an allograft biopsy prior to explantation. These 3 transplantectomies were performed on day 8, 11 and 12 after transplantation. The diagnosis was acute T-cell-mediated rejection in all 3 patients and all had urine production after transplantation. The remaining 5 transplantectomies did not show CaOx deposition, and all were explanted within 4 days after transplantation. No prior allograft biopsy was performed in these patients. The diagnosis was thrombosis in 4 of the cases and hyperacute rejection in 1 case. Two of these patients did not have urine production after transplantation and in the other 3 urine volume had been low.

The transplantectomies were not included in any of the statistical analysis performed in this manuscript.

### Graft failure

**Fig 2:**
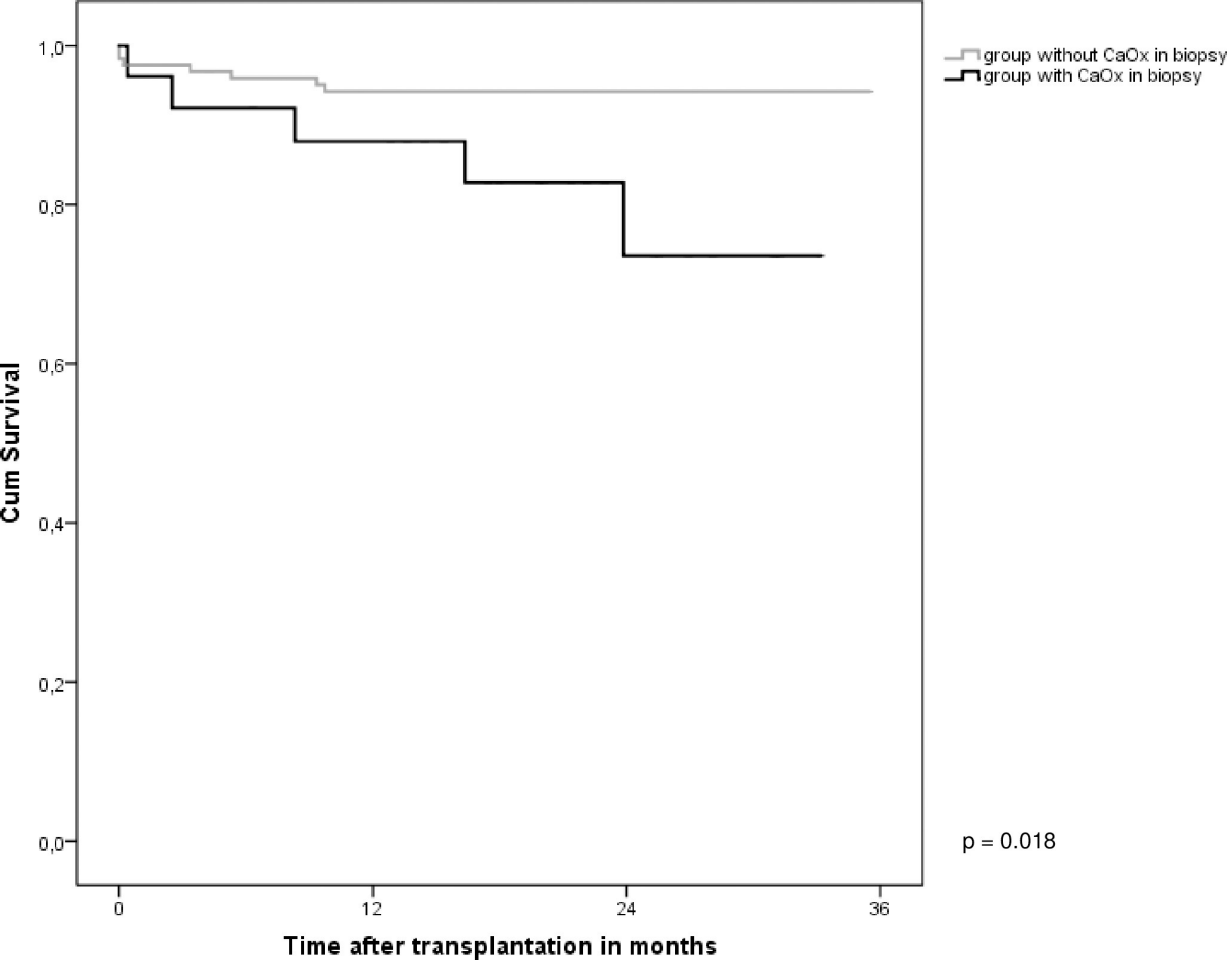
Graft survival censored for death in the group with CaOx deposition (N = 26) and the group without CaOx deposition (N = 123) within the first 3 months after transplantation (p = 0.018).

**Fig 3:**
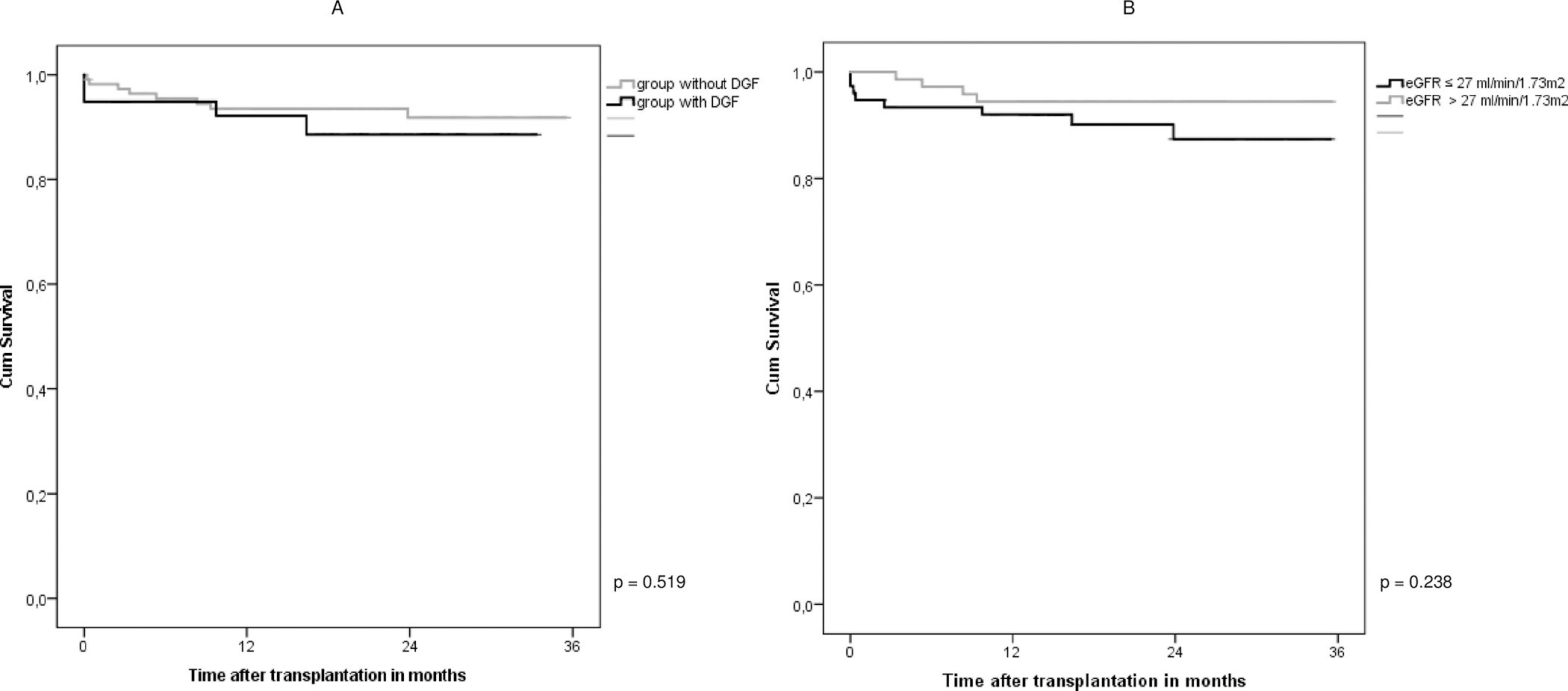
(A) Graft survival censored for death in the group with DGF (N = 39) and the group without DGF (N = 110) (p = 0.519). (B) Graft survival censored for death in the group with eGFR ≤27 ml/min/1,73m^2^ at time of first biopsy (N = 76) and the group with eGFR >27 ml/min/1,73m^2^ at time of first biopsy (N = 73) (p = 0.238).

Graft failure censored for death was worse in the group of patients with CaOx deposition in their renal allograft biopsy within the first 3 months after transplantation compared to those patients without CaOx deposition in their allograft biopsy (p = 0.018; Fig 2). In the CaOx group, 5 grafts failed (19%) vs. 7 (6%) in the group without CaOx deposition. Both DGF and eGFR ≤27 ml/min/1,73m^2^ at time of first biopsy were not associated with graft loss (p = 0.238 and p = 0.519 respectively; Fig 3A and 3B). No significant association was observed between CaOx density ≥ 0,8 deposits/ mm^2^ and graft loss (p = 0.133).

### CaOx in preimplantation renal transplant biopsies (t0)

A total of 106 t0 kidney biopsies were available for analysis. Fifty-six (53%) biopsies were from living donors and 50 (47%) from deceased donors. CaOx deposition was found in 1 t0 biopsy (0.94%) which was from a living unrelated donor. This otherwise completely healthy donor was diagnosed with his first renal stone (type unknown) 7 years after donation.

## Discussion

This study demonstrates that CaOx deposition is present in as many as 17% of the patients with a for-cause renal allograft biopsy within the first 3 months after transplantation and that these deposits are primarily recipient-derived. DGF was more common in patients with CaOx in their biopsies. In addition, the presence of CaOx was associated with a significantly lower eGFR at the time of biopsy in both univariate and multivariate analysis. As there was no significant difference in best eGFR before biopsy, this indicates that patients with CaOx deposition had a greater loss of renal function prior to biopsy.

The presence of CaOx deposits may have contributed to the impairment of renal allograft function. Risk factors for CaOx deposition are a high concentration of oxalic acid in the urine, damage to renal tubular cells, volume depletion and oliguria that leads to low tubular flow [32–33]. The CaOx crystals attach to the renal tubular cells and can cause (further) damage to the tubular cells [34–35]. This all can lead to a vicious circle of damage to the allograft due to CaOx deposition and renal function impairment.

Only a handful of other studies have examined CaOx deposition in renal allograft biopsies. Truong *et al*. reported CaOx deposition in about 4% of unselected renal allograft biopsies (13/315). These CaOx positive biopsies were performed between 4 days and 10 months after transplantation [36]. Bagnasco *et al*. observed CaOx deposition in 9% of renal allograft recipients with at least one for-cause biopsy in the first year after transplantation [30]. The study by Pinheiro *et al*. found CaOx deposition in 52.8% of renal allograft biopsies within 3 months after transplantation [37]. The percentage reported in the latter study is remarkably high in comparison to the present and previous studies. There are several possible explanations for this difference. The use of naftidrofuryl oxalate, a peripheral vasodilator that was used in that time period for the management of vascular disease, may have been responsible for the high incidence of CaOx observed by Pinheiro *et al*. Naftidrofuryl oxalate is now known to cause high serum oxalic acid concentrations [38–39]. The study by Pinheiro *et al*. was performed in Sao Paulo, Brazil. Different dietary habits between populations may play a role, while the oxalic acid content in food may also differ considerably dependent on the oxalate content of the soil where it was grown. In a very recent study, Palsson *et al*. showed comparable results to our study as they observed CaOx deposition in 19.4% (67/346) of the patients with a renal allograft biopsy within 3 months after transplantation [40].

In our study, patients with CaOx deposition significantly more often received RRT before transplantation. Both univariate and multivariate analysis confirmed an association between RRT and CaOx deposition, although the duration of RRT did not influence results. Most probably the newly transplanted kidney is exposed to higher pre-transplant oxalic acid levels in patients dependent on RRT compared to those with impaired but functioning kidneys. Previous studies showed that hemodialysis is more efficient in removing oxalic acid than peritoneal dialysis [19–20]. However, no difference in CaOx deposition between these treatment modalities was found in the present study. Possibly patients with peritoneal dialysis were treated more recently than patients treated with hemodialysis as the first mentioned is done on a daily basis. However, the number of patients included in our study might be too small to make this distinction.

Multivariate analysis showed that the time after transplantation until performance of the first allograft biopsy significantly influenced the prevalence of CaOx deposition: a longer period was associated with a higher risk of CaOx deposition. Surprisingly, donor type (living or deceased) did not influence the prevalence of CaOx deposition in the population with a biopsy obtained within 3 months after transplantation.

DGF was significantly more common among patients with CaOx deposition compared to those without (43% vs. 24%). This is consistent with the findings of Pinheiro *et al*. who found a significantly higher incidence of DGF among patients with CaOx in their renal allograft biopsy compared to those without CaOx deposition [37]. The sequence in the relationship between CaOx deposition and DGF in the renal allograft is challenging. CaOx can cause direct injury to the tubular cells which may promote the pathogenesis of DGF in the post-transplantation period [41, 42]. However, it is also possible that injury to the tubular epithelial cells and low tubular flow as a result of DGF facilitate CaOx deposition. In our study, acute tubular injury was consistently observed in the majority of biopsies with CaOx deposits.

It is remarkable that our early transplantectomies did not show CaOx deposition, while transplantectomies from day 8 onwards did. An explanation may be that in cases with early transplantectomy there was no renal function and no urine production at all after transplantation, for example due to thrombosis or hyper acute rejection.

We found that graft survival censored for death was significantly worse in patients with CaOx deposition within 3 months after transplantation compared to those without, while DGF and eGFR at time of first biopsy were not associated with graft loss. Due to the limited number of patients with graft loss during follow up, a multivariable analysis to investigate the independent causal association of CaOx deposition on graft survival could not be performed. Our graft survival results are in line with those reported by Pinheiro *et al*. who showed a 1-year renal allograft survival of 72.5% in the group with CaOx deposition and 89.1% in the group without CaOx (p = 0.013) [37].

Histological evaluation of preimplantation biopsies provides information on organ quality and can help predict short and long-term outcomes of the renal allografts [43–45]. Bagnasco *et al*. were the first to study the prevalence of CaOx deposition in t0 biopsies (n = 26) and found no CaOx deposition in any of the donor kidney biopsies [30]. Our results are in line with these findings as we only observed CaOx deposition in 0.94% of t0 kidney biopsies. These findings suggest that CaOx deposition in the renal transplant is recipient rather than donor-derived. The 106 t0 biopsies were studied in the population that donated in 2000 and 2001. No t0 biopsies were performed in the population that donated to our patient cohort transplanted in 2014 and 2015. However, it is unlikely that the results will be different in this specific donor population.

Unfortunately, plasma oxalic acid levels pre-transplantation were not available in the present study. Additional studies are needed on the relationship between plasma oxalic acid levels pre-transplantation and the risk for CaOx deposition and inferior graft survival post-transplantation. This could aid in differentiating those patients that might benefit from preventive treatment to reduce plasma oxalic acid levels pre- and post-kidney transplantation. Avoidance of food products with high oxalic acid content and/or adequate hemodialysis treatment in the direct pre-transplant phase might prevent unnecessary CaOx deposition in the new renal allograft.

In this study, we showed that CaOx deposition coincides with other causes of renal function impairment in the setting of decreased urinary flow such as DGF. We showed that CaOx deposition in the renal allograft biopsy shortly after transplantation is common and associated with inferior long-term graft outcome. Therefore, we strongly advise the systematic examination of all for-cause renal allograft biopsies by polarized light for CaOx deposition.

## Supporting information

S1 File The database used in this study. (XLSX)
